# The complete mitochondrial genome of the Diqing pig

**DOI:** 10.1080/23802359.2018.1522978

**Published:** 2018-10-27

**Authors:** Yuan Cai, Qianyun Ge, Guoshun Chen, Tiantuan Jiang, Qiaoli Yang, Xiaoyu Huang, Shengguo Zhao

**Affiliations:** College of Animal Science and Technology, Gansu Agricultural University, Lanzhou, China

**Keywords:** Diqing pig, mitochondrial DNA, NJ Phylogenetic tree

## Abstract

Diqing pig is one of the famous native breed in China. In this work, we reported the complete mitochondrial genome sequence of the Diqing pig in Hunan Province for the first time. The total length of the mitogenome is 16,720 bp. The NJ phylogenetic tree analysis showed that Diqing pig together with Chinese animals, and the farthest genetic distance from Landrace, has the closest genetic distance to Ganzi pig.

Diqing pig native to the highlands of northwest China Yunnan Diqing (Altitude 3000 m ∼ 4000 m, East longitude 94° ∼ 102°,Northern latitude 26° ∼ 34°）and the sample was stored in the laboratory of Gansu Agricultural University. These animals allowed them to adapt to extreme conditions such as hypoxia (Li et al. 2013). This study first reported complete mitochondrial DNA sequence of Diqing pig and compared with other breeds. Mitochondrial DNA was extracted using the EasyPure Kit of Genomic DNA (Transgen Biotech, Beijing, China). PCR was carried out with 24 pairs of primers designed according to the Landrace pig (GenBank accession number NC_000845.1) (Xu et al. 2015). DNA sequence was analyzed using MEGA7 software (https://www.ncbi.nlm.nih.gov/pubmed/27004904) (Kumar et al. 2016).

The total length of the mitogenome is 16,720 bp. With the base composition of 34.7% for A, 25.8% for T, 26.2% for C, 13.3% for G, respectively, and an A + T (60.5%)-rich feature. It is made up of 2 ribosomal RNA genes, 13 protein-coding genes, 22 transfer RNA genes, and 1 D-loop region. The arrangement of these genes was the same as that found in the Landrace pig. The initiation codon for ND2, ND3, and ND5 is ATA, for ND4L is GTG, for ND6 is TTA and the rest of the proteins is ATG. Of all these genes were found in 7 overlaps and 12 spaces in the length of 1–43 bp. These genes had five types of termination codons, including CAT for ND1 and ND6, ACT for ND2, TAA for COX1, ATP8, ATP6, ND4L, ND5, AGA for Cytb, and an incomplete termination codon T- for COX2, COX3, ND3, ND4. The D-loop region locates between tRNAPro and tRNAPhe with a length of 1284 bp. The analysis found that Diqing pig together with 25 Chinese animals, Large White, and Landrace has the farthest distance from Landrace (0.003) and nearest distance to Ganzi pig. Chinese northeast wild boar forms a distinct branch. European animals form a distinct outgroups (Figure 1).

**Figure 1.**
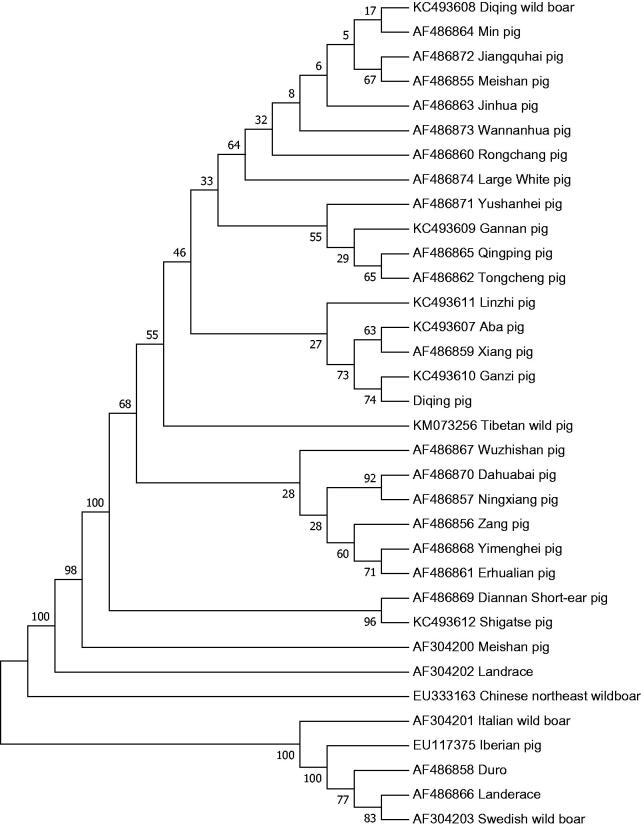
Phylogenetic trees based on the complete mitochondrial genome by Neighbor-joining analysis.

The evolutionary history was inferred using the Neighbor-Joining method (Saitou and Nei 1987). The optimal tree with the sum of branch length = 0.02743855 is shown. The percentage of replicate trees in which the associated taxa clustered together in the bootstrap test (1000 replicates) are shown next to the branches (Felsenstein 1985). The tree is drawn to scale, with branch lengths in the same units as those of the evolutionary distances used to infer the phylogenetic tree. The evolutionary distances were computed using the Kimura 2-parameter method (Kimura 1980) and are in the units of the number of base substitutions per site. The rate variation among the sites was modeled with a gamma distribution (shape parameter = 1). The analysis involved 34 nucleotide sequences. Codon positions included were 1st + 2nd + 3rd + Noncoding. All the positions containing gaps and missing data were eliminated. There were a total of 15,366 positions in the final dataset. Evolutionary analyses were conducted in MEGA7 (Kumar et al. 2016).
